# Analysis of prognostic value of lactate metabolism-related genes in ovarian cancer based on bioinformatics

**DOI:** 10.1186/s13048-024-01426-z

**Published:** 2024-05-22

**Authors:** Jinrui Sun, Qinmei Feng, Yingying Xu, Ping Liu, Yumei Wu

**Affiliations:** 1 Department of Gynecologic Oncology, Beijing Obstetrics and Gynecology Hospital, Beijing Maternal and Child Health Care Hospital, Capital Medical University, Beijing, 100006 China; 2 Department of Gynecology, Shanxi Provincial People’s Hospital, Taiyuan, 030001 Shanxi Province China

**Keywords:** Lactate metabolism-related genes, Ovarian cancer, Prognostic model, qRT-PCR

## Abstract

**Background:**

Recent studies have provided evidence supporting the functional role and mechanism of lactate in suppressing anticancer immunity. However, there is no systematic analysis of lactate metabolism-related genes (LMRGs) and ovarian cancer (OV) prognosis.

**Results:**

Six genes (CCL18, CCND1, MXRA5, NRBP2, OLFML2B and THY1) were selected as prognostic genes and a prognostic model was utilized. Kaplan-Meier (K-M) and Receiver Operating Characteristic (ROC) analyses were further performed and indicated that the prognostic model was effective. Subsequently, the neoplasm_cancer_status and RiskScore were determined as independent prognostic factors, and a nomogram was established with relatively accurate forecasting ability. Additionally, 2 types of immune cells (Central memory CD8 T cell and Immature B cell), 4 types of immune functions (APC co inhibition, DCs, Tfh and Th1 cells), 9 immune checkpoints (BTLA, CTLA4, IDO1, LAG3, VTCN1, CXCL10, CXCL9, IFNG, CD27) and tumor immune dysfunction and exclusion (TIDE) scores were significantly different between risk groups. The expression of 6 genes were verified by quantitative Real-Time Polymerase Chain Reaction (qRT-PCR) and the expression of 6 genes were higher in the high-grade serous carcinoma (HGSC) samples.

**Conclusion:**

A prognostic model related to lactate metabolism was established for OV based on six genes (CCL18, CCND1, MXRA5, NRBP2, OLFML2B and THY1) that could provide new insights into therapy.

## Introduction

Ovarian cancer (OV) is a malignant tumor that seriously threatens women’s health, with the third highest incidence and the highest mortality rate among all malignant tumors of the female reproductive system [1]. Due to the lack of effective screening strategies, ovarian cancer exhibits late onset of clinical symptoms, so about 60% of ovarian cancer patients are already advanced at the time of diagnosis [2]. Although surgery, chemotherapy, biological therapy, and gene therapy are widely used in the treatment of OV, the 5-year survival rate of OV patients is still as low as 35–38% [1, 3]. Therefore, exploring the pathogenesis of OV is of great significance for the early detection, diagnosis and treatment [4, 5].

Numerous studies have shown that lactic acid plays an important role in suppressing anti-cancer immunity [6]. Choi SY et al. indicated that lactic acid and the lactic acid-generating metabolic microenvironment have a wide range of effects on cancer, such as: angiogenesis, local invasion, distant metastasis and anti-cancer immune response. Cancer-generated lactic acid could thus be viewed as a critical, immunosuppressive metabolite in the tumour micro-environment [7]. Yuanyuan Zhou et al. also revealed that the anti-tumor effect in OV could be exerted via antagonize the Warburg effect by inhibiting glucose consumption and lactic acid production [8]. Rahul Bhattacharya et al. also suggested that the lactate metabolism-related gene FGF9 can induce the invasion of OV cells by enhancing the expression of VEGF-A/VEGFR2 [9]. In addition, Jiangdong Xiang et al. found that the increased expression of LDH, a key enzyme that regulates lactate metabolism, is associated with high metastasis, high invasion and low survival rate of OV [10]. However, few studies have comprehensively analyzed the relationship between lactate metabolism related genes (LMRGs) and the prognosis of OV.

Bioinformatics technology is a promising instrument for understanding the mechanisms of tumorigenesis and progression, and has been widely used in the study of a wide range of tumors [11–14]. In this study, we downloaded the OV RNA-sequencing data and clinical data from the TCGA database, classified OV patients according to the 15 lactate metabolism-related genes retrieved from the MSigDB database, and identified the genes associated with OV. Process-related differentially expressed genes constructed a prognostic risk model, which could provide potential targets for the clinical diagnosis and prognosis of OV.

## Results

### Survival analysis and correlation analysis of clinical features between patients with 2 clusters

**Fig. 1:**
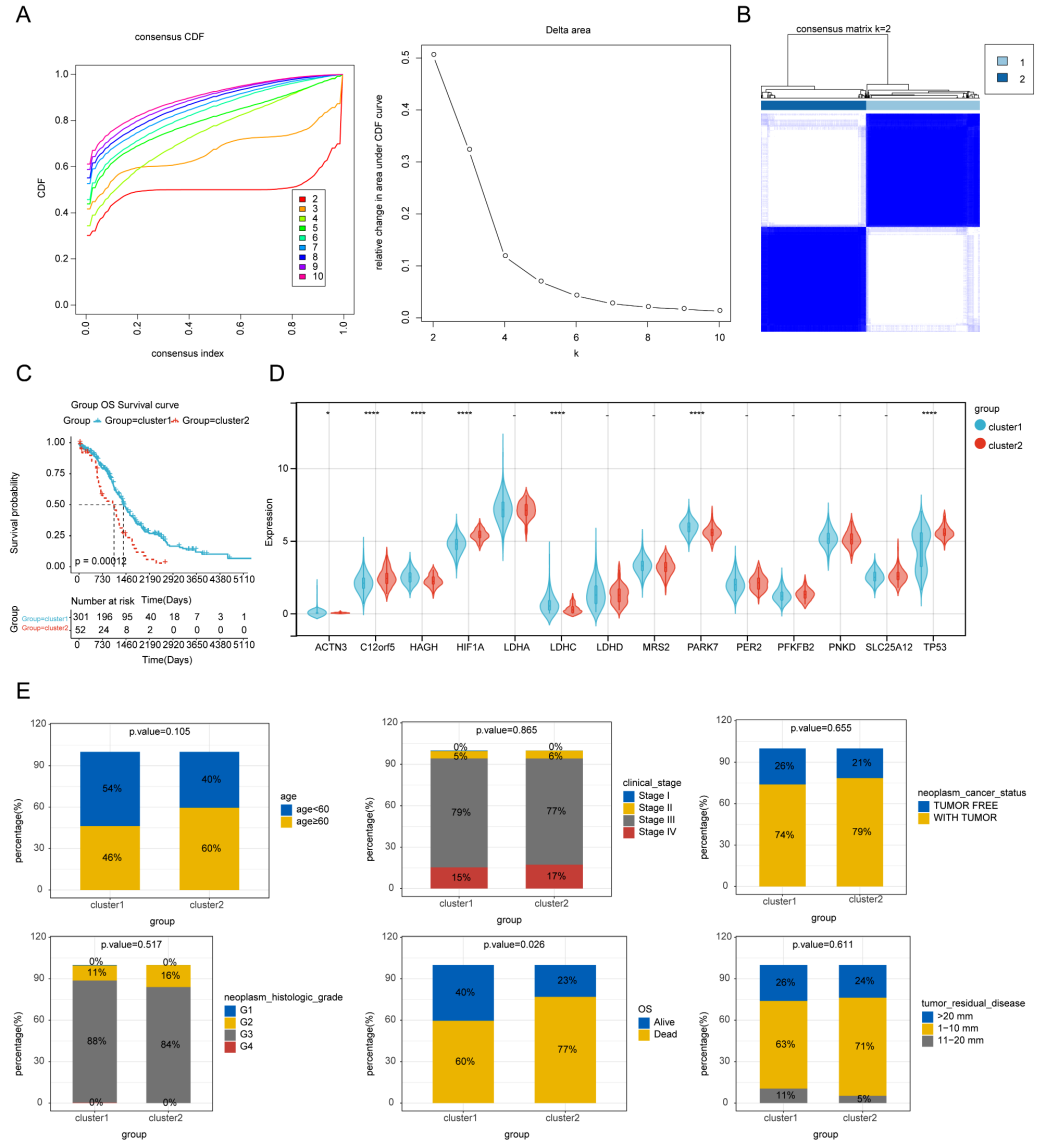
Lactate metabolism related genes (LMRGs) could classify the clinical and molecular features of ovarian cancer. **(A)** Consensus clustering cumulative distribution function (CDF) for k = 2 to k = 10 (up). Relative change in area under CDF curve according to various k values (down). **(B)** Consensus clustering matrix of 353 OV samples from TCGA dataset for k = 2. **(C)** Survival analysis of OV patients in Cluster 1 and 2 in TCGA cohort. **(D)** Violin chart of the expression levels of LMRGs in two clusters. *p < 0.05; ****p < 0.0001. (E) Stacking diagrams of the correlations of clinical features between two clusters

The The Cancer Genome Atlas (TCGA) samples were divided into 2 clusters according to the K = 2 (Fig. 1A and B). The Kaplan-Meier (K-M) curves was shown that there is a significant difference in survival between patients with cluster1 and cluster2 (*p* = 0.00012) (Fig. 1C), with cluster1 patients having a good prognosis than cluster2 patients. 7 lactate metabolism-related genes (LMRGs) were different between the 2 clusters, containing ACTN3, C12orf5, HAGH, HIF1A, LDHC, PARK7 and TP53 (Fig. 1D). Furthermore, the correlation analysis of clinical features found that the OS was significantly associated with different clusters (Fig. 1E).

### Analysis of immune infiltration among different clusters

**Fig. 2:**
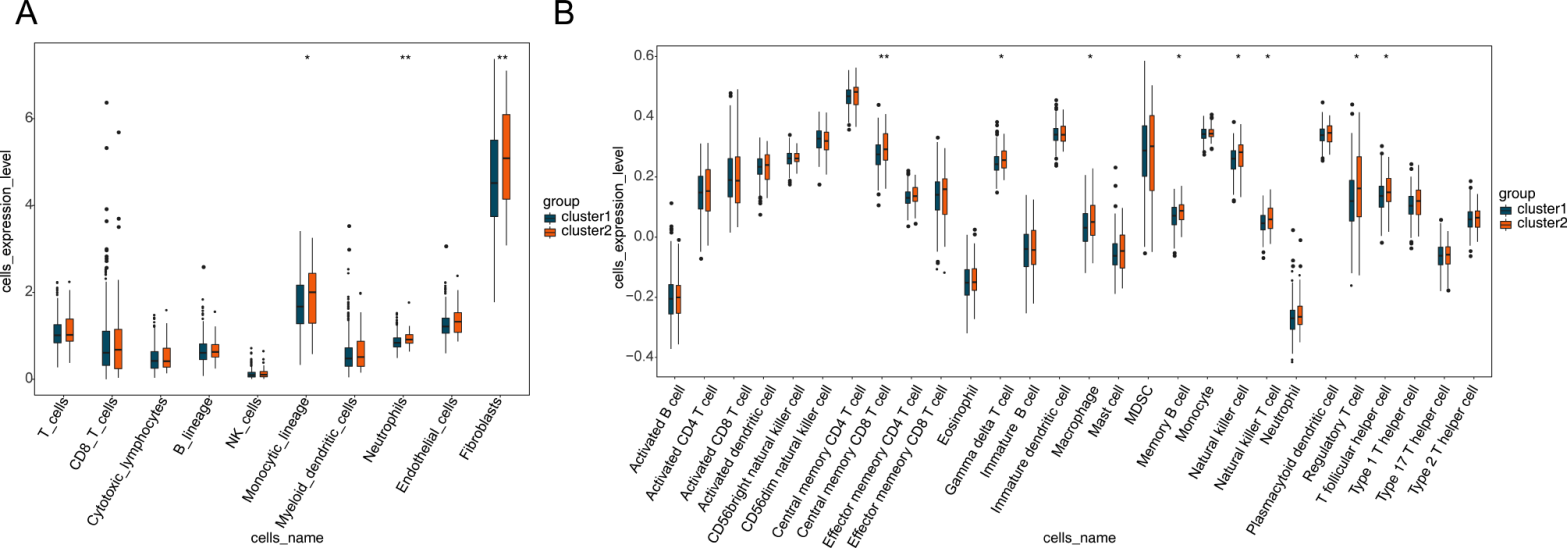
Immune infiltration analysis. **(A)** Box plot of the expression levels of 10 immune cell types between two clusters by MCPcounter algorithm. *p < 0.05; **p < 0.01. **(B)** The expression levels of 28 immune cell types in two clusters were assessed by ssGSEA. The differences in two groups were compared using Wilcox test. *p < 0.05, **p < 0.01

There are 3 immune cells with differences among different clusters in the MCPcounter algorithm (monocytic lineage, Neutrophils and Fibroblasts) (Fig. 2A). 8 immune cells with differences among different clusters in the ssGSEA algorithm (Central memory CD8 T cell, Gamma delta T cell, Macrophage, Memory B cell, Natural killer cell, Natural killer T cell, Regulatory T cell and T follicular helper cell) (Fig. 2B).

### Differential gene analysis

**Fig. 3:**
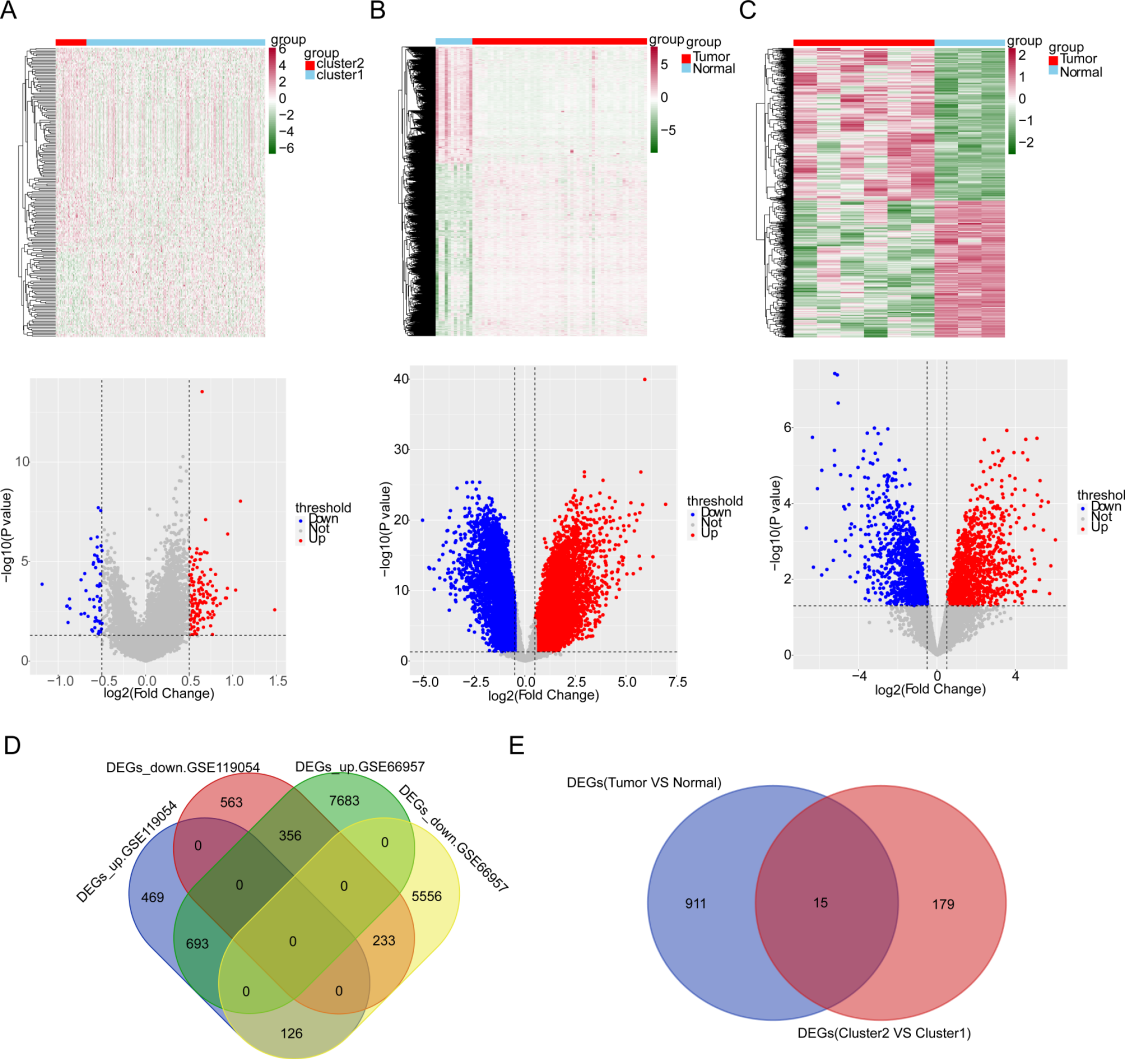
Identification of 15 key genes. **(A)** Volcano plot showed 137 up-regulated (red) and 57 down-regulated genes (blue) in two clusters, and the heatmap showed their expresion levels. **(B)** Volcano plot showed 8732 up-regulated (red) and 5915 down-regulated genes (blue) between OV and normal samples in GSE66957 dataset, and the heatmap showed their expression levels. **(C)** Volcano plot showed 1,288 up-regulated and 1,152 down-regulated genes between OV and normal samples in GSE119054 dataset, and the heatmap showed their expression levels. **(D)** Venn diagram showed 926 overlapping genes by overlapping differentially expressed genes (DEGs) from GSE66957 and GSE119054 datasets. **(E)** 15 key genes were obtained by intersecting 194 DEGs between two clusters and 926 overlapping genes

A total of 194 differentially expressed genes (DEGs) were acquired from the cluster2 VS cluster1 (137 up-regulated and 57 down-regulated genes) (Fig. 3A). In GSE66957 and GSE119054 datasets, 14,647 DEGs (8,732 up-regulated and 5,915 down-regulated genes) (Fig. 3B) and 2,440 DEGs (1,288 up-regulated and 1,152 down-regulated genes) (Fig. 3C) were acquired between tumor and normal, respectively. Subsequently, 926 overlapping DEGs (693 up-regulated and 233 down-regulated genes) were acquired from the GSE66957 and GSE119054 datasets (Fig. 3D), and 15 key genes were obtained from the 926 overlapping DEGs and 194 DEGs (Fig. 3E).

### Construction and validation of 6-genes prognostic model

**Fig. 4:**
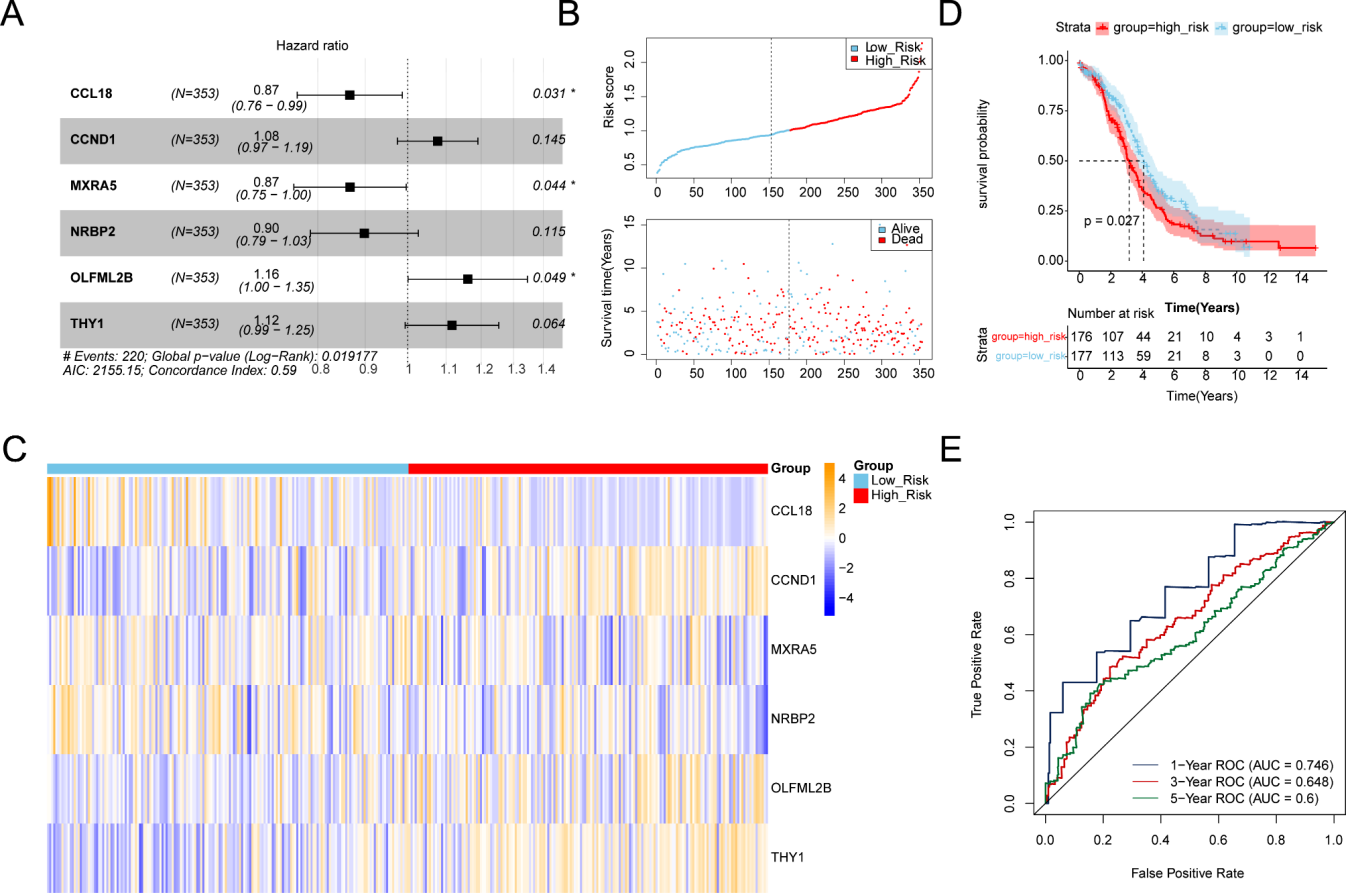
Construction of a 6-genes prognostic model in the training set. **(A)** Forest plot of six prognostic genes. **(B)** The heatmap of the expression of six model genes in the high- and low-risk groups. **(C)** Kaplan-Meier survival curves of the training group using 1.006764082 as the cutoff value. **(D)** Receiver Operating Characteristic (ROC) curves for 1-, 3-, and 5-year survival in the training set (AUC: 0.746, 0.648, 0.6)

**Fig. 5:**
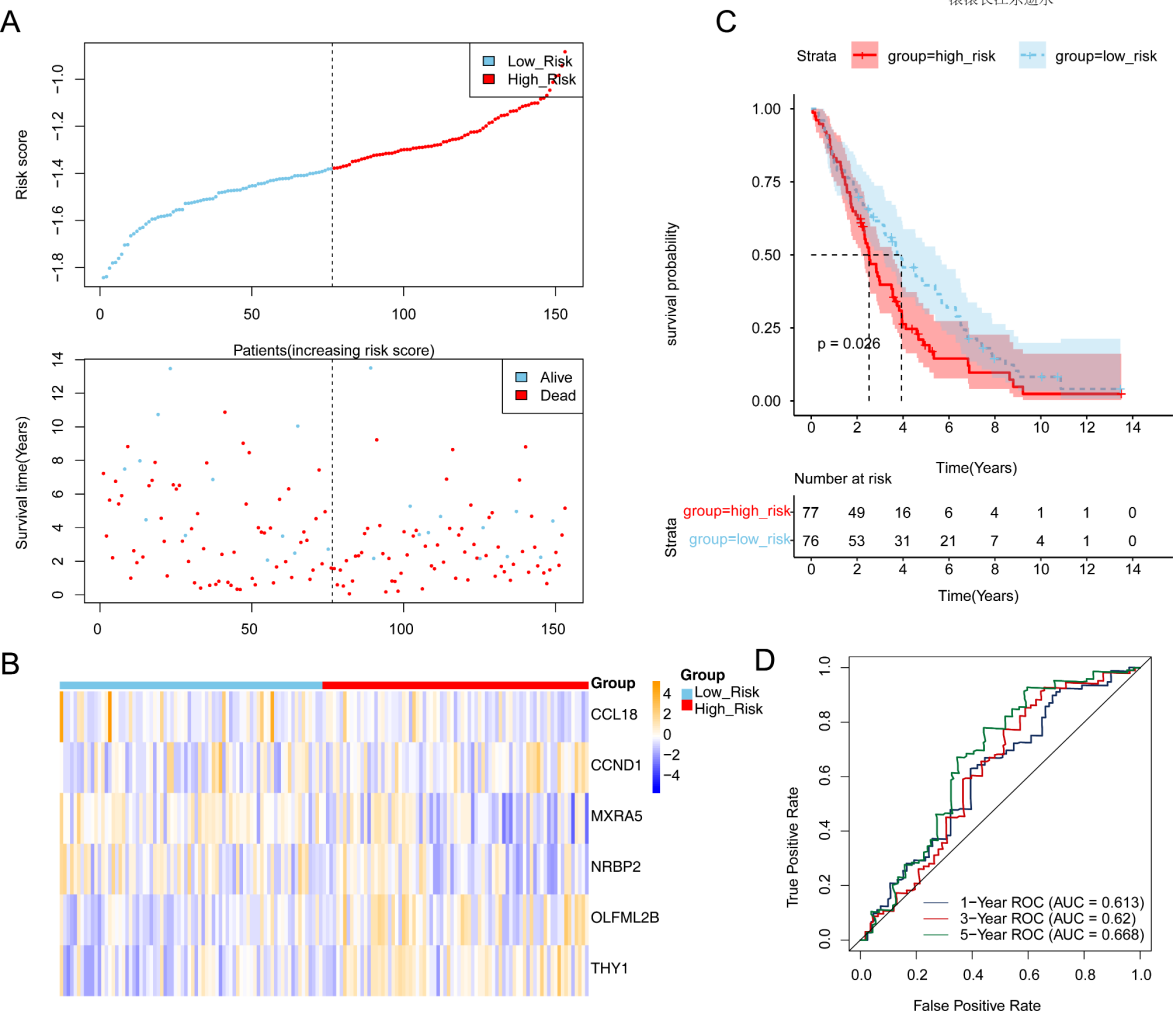
Validation of the 6-genes prognostic model in the validation set. **(A)** Forest plot of six prognostic genes. **(B)** The heatmap of the expression of six model genes in the high- and low-risk groups. **(C)** Kaplan-Meier curves for the overall survival of lung adenocarcinoma patients with high- and low-risk groups. **(D)** Receiver Operating Characteristic (ROC) curves for 1-, 3-, and 5-year survival in the training set (the area under ROC curve (AUC): 0.613, 0.62, 0.668)

Among the 15 key genes, 6 genes (CCL18, CCND1, MXRA5, NRBP2, OLFML2B and THY1) were selected (Fig. 4A) as prognostic genes. The 353 patients of the training set were grouped into high- (176) and low-risk (177) groups based on the median risk scores (1.006764082) (Fig. 4B). The expression of CCL18, MXRA5 and NRBP2 were negative correlated with risk scores, and the expression of CCND1, OLFML2B and THY1 positive correlated with risk scores (Fig. 4C). The overall survival (OS) (*p* = 0.027) of risk groups were different (Fig. 4D). The AUCs of 1-, 3-, 5-year were higher 0.6, indicating that the prognostic model was effective (Fig. 4E). Additionally, the prognostic model was verified in the GSE26712 dataset, and the results of gene expression, K-M analysis and Receiver Operating Characteristic (ROC) analysis were consistent with the training set (Fig. 5A-D).

### Risk model was associated with neoplasm cancer status and survival status

**Fig. 6:**
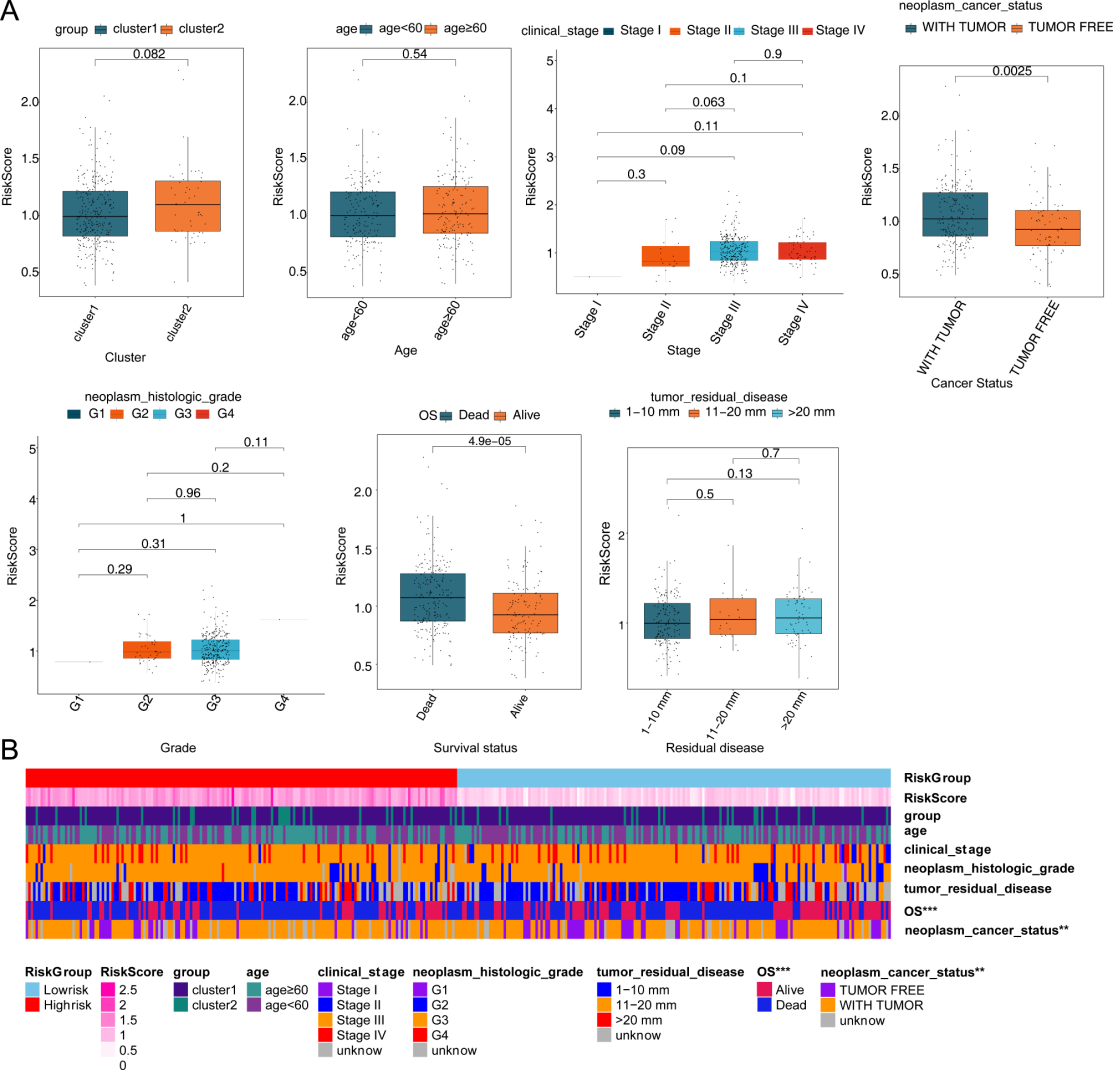
Correlation between risk score and different clinical features for the training set. **(A)** Box plot of the correaltions between risk score and clinical features. **(B)** Heatmap of the associations between risk scores and clinicopathological features. **p < 0.01; ***p < 0.001

To investigate the correlation between risk models and different clinicopathological features, the correlation was performed on the Fig. 6A-B. The results indicated that the risk model was associated with neoplasm cancer status and survival status.

### Independent prognostic analysis of risk models and construction of nomogram

**Fig. 7:**
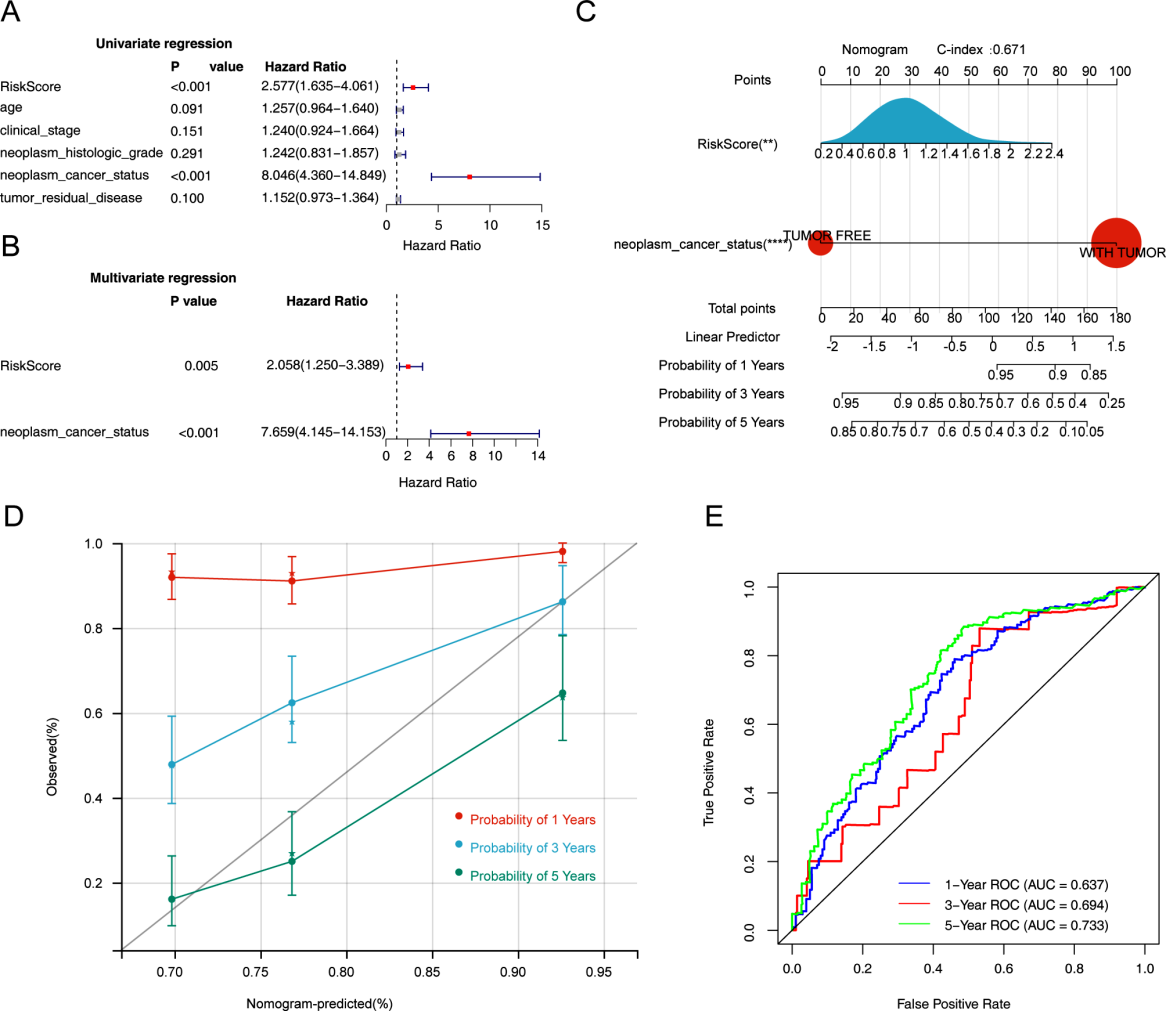
Establishment of a prognostic nomogram. **(A)** Univariate Cox analysis of risk score and clinical features. **(B)** The multivariate analysis showed the independent prognostic factors. **(C)** A prognostic nomogram based on independent prognostic factors. **p < 0.01; ****p < 0.0001. **(D)** Calibration plot showed that nomogram-predicted survival probabilities corresponded closely to the observed proportions. **(E)** ROC curve showed that the nomogram could accurate forecasting the survival probability at 5-year survival

Cox analyses were utilized to selected independent prognostic factors. The neoplasm cancer status and RiskScore were screened by univariate Cox analysis (*p* < 0.05) (Fig. 7A). Subsequently, the neoplasm cancer status and RiskScore were determined as independent prognostic factors (*p* < 0.05) (Fig. 7B), and a nomogram was established (C-index = 0.671) (Fig. 7C). The calibration curve and ROC curve indicated that the constructed prediction model could be used as an effective model with relatively accurate forecasting ability (Fig. 7D and E).

### Immune-related analysis between the risk groups

**Fig. 8:**
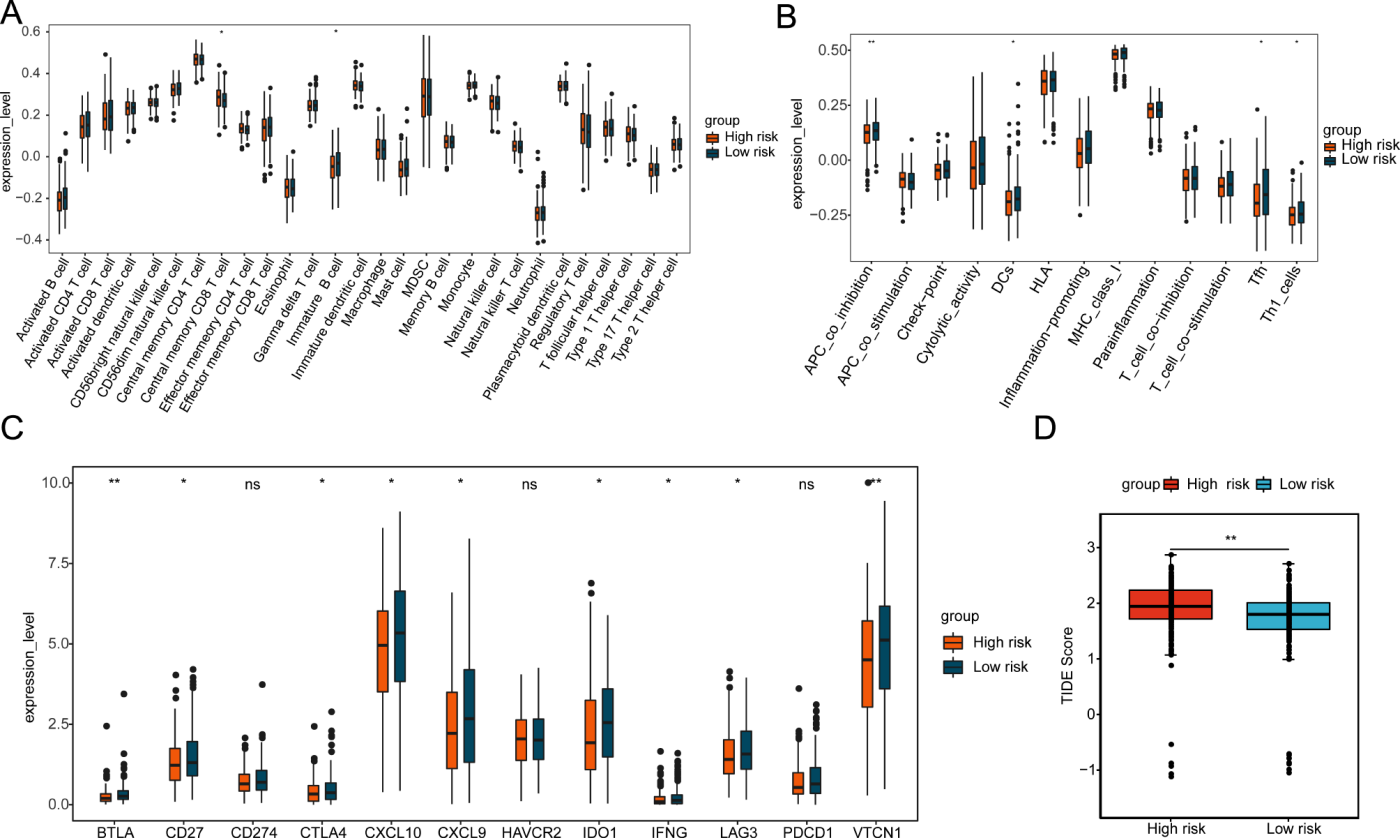
The characteristics of immune infiltration based on six-gene model in the training set. **(A)** Box plot of the expression levels of 28 immune cell types between high- and low-risk groups by ssGSEA. *p < 0.05. **(B)** The expression levels of 13 immune cell types between high- and low-risk groups. *p < 0.05; **p < 0.01. **(C)** The expression levels of 12 immune checkpoints between high- and low-risk groups. ns, not significant; *p < 0.05; **p < 0.01. **(D)** TIDE scores in high- and low-risk groups. **p < 0.01

Since the immune microenvironment is closely related to tumorigenesis and progression, we performed immune infiltration analysis and assessed the effect of immunotherapy response. The results revealed that the expression of 2 types of immune cells (Central memory CD8 T cell and Immature B cell) (Fig. 8A), 4 types of immune functions (APC co inhibition, DCs, Tfh and Th1 cells) (Fig. 8B), 9 immune checkpoints (BTLA, CTLA4, IDO1, LAG3, VTCN1, CXCL10, CXCL9, IFNG, CD27) (Fig. 8C) were significantly different between high- and low-risk groups. In addition, the tumor immune dysfunction and exclusion (TIDE) score (Fig. 8D) *was* significantly higher in the high-risk group than in the low-risk group, indicating that the high-risk group was less sensitive to immunotherapy.

### Functional enrichment analysis

**Fig. 9:**
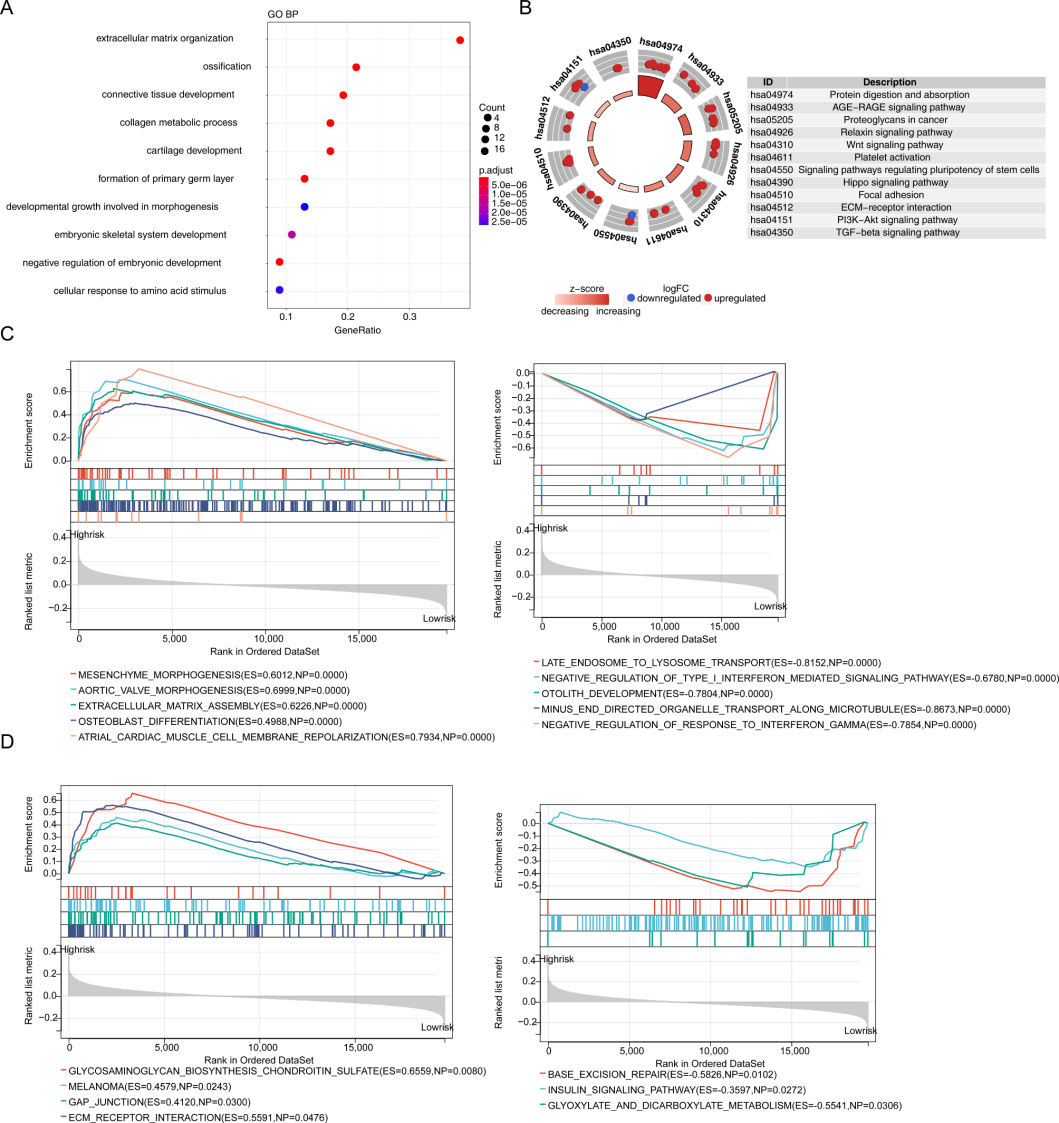
Functional enrichment analysis of 51 DEGs between high- and low-risk groups. **(A)** The bubble plot of the top 10 GO BP terms were enriched for 51 DEGs. **(B)** KEGG functional enrichment analysis for 51 DEGs. **(C)** The top 5 GO-BP terms enriched in high- (left) and low-risk (right) groups by GSEA analysis, respectively. **(D)** The KEGG pathways enriched in high- (left) and low-risk (right) groups by GSEA analysis.DEGs: differentially expressed genes; BP: biological process; GSEA: gene set enrichment analysis; GO: gene notology; KEGG: Kyoto Encyclopedia of Genes and Genomes

There were 51 DEGs in High_risk VS Low_risk. These 51 DEGs were enriched in 152 Gene Ontoloty (GO)-biological process (BP) terms and 12 KEGG pathways (Fig. 9A and B). These GO-BP terms and Kyoto Encyclopedia of Genes and Genome (KEGG) pathways were related with immune functions and pathways, such as negative regulation of T cell proliferation, negative regulation of immune system process, positive regulation of humoral immune response, negative regulation of T cell receptor signaling pathway and PI3K-Akt signaling pathway.

Moreover, the different enrichment GO terms and KEGG pathways of the High_risk VS Low_risk were related various cell signal transduction and metabolism, such as aortic valve morphogenesis, late endosome to lysosome transport, negative regulation of B cell mediated immunity, insulin signaling pathway, glycosaminoglycan biosynthesis chondroitin sulfate, and glyoxylate and dicarboxylate metabolism (Fig. 9C and D).

### Quantitative real-time polymerase chain reaction (qRT-PCR)

**Fig. 10:**
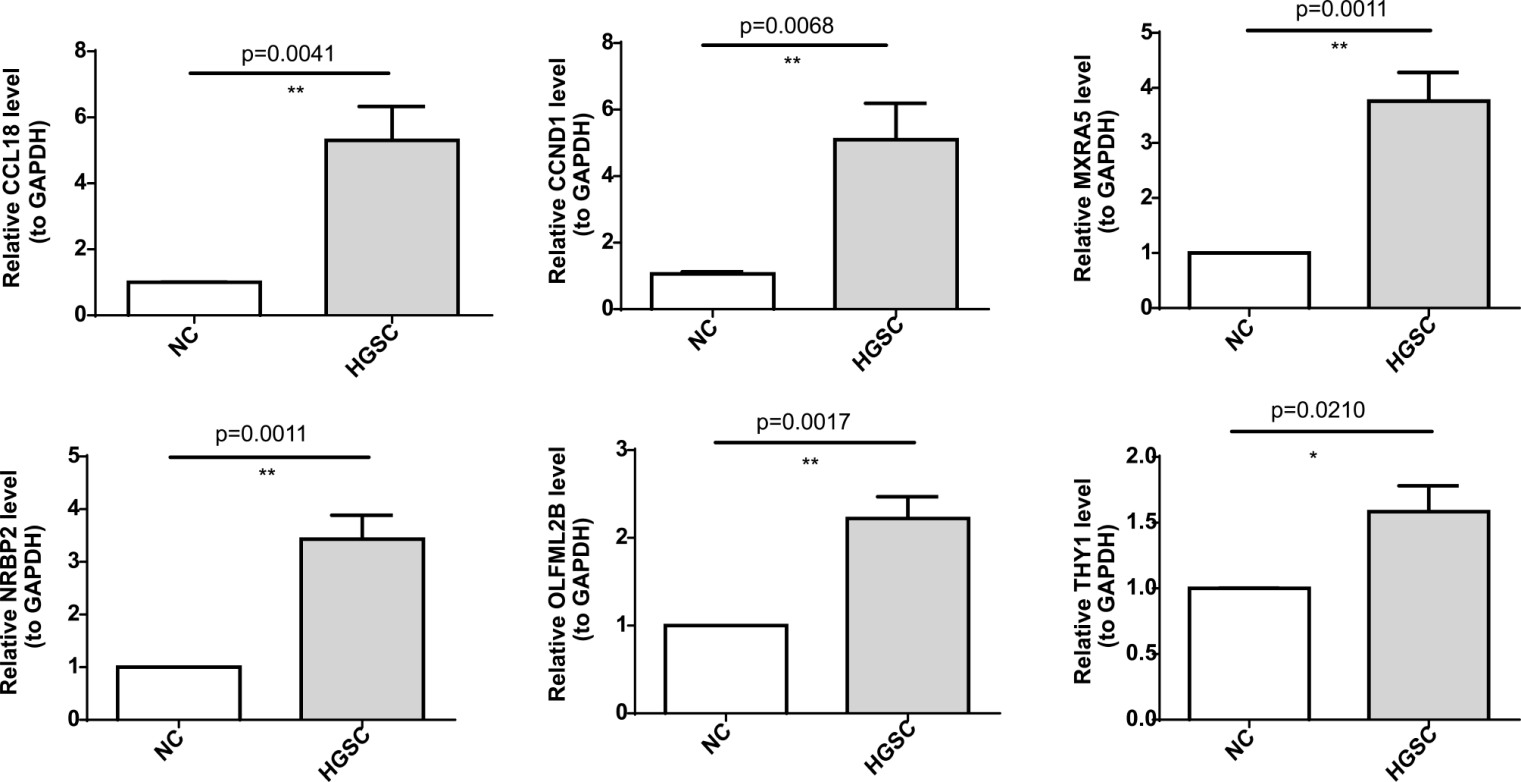
Validation of the expression of six prognostic genes by qRT-PCR. *p < 0.05, **p < 0.01

The expression of 6 genes (CCL18, CCND1, MXRA5, NRBP2, OLFML2B and THY1) were verified by qRT-PCR (Fig. 10). In addition, the expression of 6 genes were higher in the high-grade serous carcinoma (HGSC) samples.

## Discussion

In recent years, great progress has been made in identifying useful biomarkers in carcinoma and para-carcinoma tissue for diagnosis, prognosis prediction, and treatment of cancer. In this study, we first downloaded the mRNA transcriptome and clinical data of OV from the public database, OV patients were divided into two subtypes through consensus clustering based on the expression matrix of genes related to lactate metabolism. These results suggest a significant correlation between survival status and the different subtypes. Additionally, seven lactate metabolism-related genes, ACTN3, C12orf5, HAGH, HIF1A, LDHC, PARK7, and TP53, were significantly different in different subtypes. In this study, a total of 15 key genes related to the occurrence and development of OV were screened, of which 6 genes, CCL18, CCND1, MXRA5, NRBP2, OLFML2B, and THY1, were identified as prognostic biomarkers for OV, including only NRBP2 was down-regulated in cluster2 OV tissues. Related literature showed that CCL18, CCND1, MXRA5, and THY1 were up-regulated in OV tissues [15–18], which is consistent with our research results. Although there is no study on the expression of OLFML2B and NRBP2 in OV, Houriiyah Tegally et al. showed that OLFML2B is over-expressed in gastric cancer and other cancers [19], Zhiyu Li et al. also showed that NRBP2 was significantly down-regulated in breast cancer tissues [20]. These studies have verified the reliability of our results.

The Cox regression analysis results showed that CCL18, MXRA5, and NRBP2 were protective factors for OV, while CCND1, OLFML2B, and THY1 were risk factors. Na Li et al. also constructed an OV risk model based on 5 biomarkers, among which CCL18 is also a protective factor [21]. Cecile Chenivesse et al. showed that CCL18 is a cytokine that exhibits chemotactic activity on various immune cells such as T cells, CD4 T cells, and CD8 T cells, and plays a role in humoral and cell-mediated immune responses [22]. In our study, there were significant differences in central memory CD8 T cells and immune B cells between high and low-risk groups, which may be potentially related to the over-expression of CCL18. Leilei Liang et al. also divided OV into two subtypes based on 97 invasion-related genes, obtained a risk model constructed from 6 biomarkers, and proved that high expression of MXRA5 was associated with low-risk and had a protective effect [23]. NRBP2 is one of the pseudokinases discovered during neural differentiation gene screening, which can inhibit the progression of various cancers such as medulloblastoma and hepatocellular carcinoma [24]. Zhiyu Li et al. have characterized NRBP2 overexpressing in breast cancer, revealing that such overexpression significantly inhibited cell proliferation and invasion, and inhibited epithelial-mesenchymal transition in cells in vitro. Conversely, knockdown of NRBP2 reversed these effects. They further illustrated that the anti-tumor effect of NRBP2 may be mediated by the AMPK/mTOR pathway [20]. CCND1, also known as cyclin D1, can sense the signal stimulation inside and outside the cell, bind and activate the cyclin-dependent kinase CDK4/6 to start the cell cycle, and is considered a mitotic cell sensor. Its abnormal expression will lead to cell cycle disorder and cellular dysfunction [25]. In addition, J Dai et al. further demonstrated that cisplatin treatment could mitigate the overexpression of CCND1 in OV cells [16]. Besides, Harindra R Abeysinghe et al. proved that the expression of THY1 is associated with OV tumor suppression through mouse experiments, signifying a positive prognostic factor for OV [26]. These studies have verified the reliability of the risk model based on the above six biomarkers. After that, based on the neoplasm cancer status score and risk score, the nomogram was established to predict the 1-, 3-, 5-year survival rates of OV patients, which would further embody the use of the prognostic model in clinical practice.

The results of the immune microenvironment of the high- and low-risk groups showed that compared with the low-risk group, 2 types of immune cells, containing central memory CD8 T cell, immature B cell, 4 kinds of immune functions including APC co-inhibition, dendritic cells, Tfh and Th1 cell functions, and 9 immune checkpoints including BTLA, CTLA4, IDO1, LAG3, VTCN1, CXCL10, CXCL9, IFNG, and CD27 were significantly decreased in the high-risk group. Tfh is the specialized provider of T cells to help B cells and essential for the development of memory B cells [27]. The APCs are a group of cells that specialize in presenting antigens to T cells, of which dendritic cells, macrophages, and B cells are the main cell types [28, 29]. In addition, DEGs between high- and low-risk groups were associated with T cell proliferation and T cell receptor signaling pathway. During the T-cell immune response, T-cell proliferation and T-cell receptor signaling pathways are important in triggering and directing T-cell activation and expansion. Thus, immune cells, especially T and B cells, are associated with the onset and development of OV, which will provide new insights into the immunotherapy in OV. Related studies have suggested that CCL18 can induce the activation of immune B cells [30], and regulatory factors in immune B cells can regulate the expression of the THY1 gene [31]. In addition, Mirco Friedrich showed that memory CD8 T cells may pass through a certain signal transduction that drives the differentiation of immune B cells to CD4 T cells [32], which may explain the decrease of immune B cells and the increase of memory CD8 T cells in the high-risk group. Experiments by Daniele Fanale et al. showed that the low expression of PD-1, PD-L1, BTN3A1, pan-BTN3As, BTN2A1, and BTLA can be used as markers for the diagnosis of advanced high-grade serous OV [33], and Xiazi Nie et al. also proved that immune checkpoints, CTLA4, IDO1, and LAG3 are all associated with poor prognosis in OV [34], in addition, CXCL10 and CD27 infiltration is associated with anti-tumor immunity and treatment response in OV subtypes [35, 36].

Finally, we performed functional enrichment analysis on the 51 DEGs in the high- and low-risk group. The results showed that these DEGs were mainly enriched in the inhibition of T cell proliferation and T cell receptor signaling pathway, PI3K Akt signaling pathway, ECM receptor interaction, focal adhesion, and multiple cell growth and development pathways. Relevant studies have suggested that OLFML2B may affect various cancer and immune-related pathways, such as ECM receptor interaction, focal adhesion, and transendothelial migration of leukocytes [19]. The tumor cells interact with various components in the ECM through their surface receptors After adhesion, protein-degrading enzymes are activated or secreted to degrade the matrix, thereby forming a localized dissolution zone and facilitating a channel for tumor cell metastasis. The enzymes that are more concerned in this process are mainly serine proteases and metalloproteases [37]. In this study, these differential genes associated with OV progression were also enriched in multiple pathways such as ECM receptor interaction, focal adhesion, collagen metabolic process, and cell response to amino acid stimulation. Negative prognostic genes such as OLFML2B may promote the adhesion of OV cells to certain components in the ECM through their surface receptors and activate the expression of metalloproteinases to degrade the matrix, thereby promoting cancer progression. Meng Li et al. showed that DDTC could effectively inhibit cell growth through the PI3K/AKT/mTOR signaling pathway, thereby inhibiting the development of OV [38]. Similarly, our study also showed that the functions of genes like CCND1, COL1A1, COL1A2, LPAR3, etc. are mainly enriched in PI3K-A, TGF-β, Hippo signaling pathways. The TGF-β signaling pathway plays a key role in the cell and tissue growth, development, and differentiation, exerting important regulatory effects on cell proliferation, interstitial production, differentiation, apoptosis, embryonic development, etc. The Hippo signaling pathway is recognized as a growth-inhibitory signaling pathway, associated with oxidative phosphorylation [39]. Obviously, these cytokines promoting OV cell growth affect the development of OV through the PI3K-Akt/TGF-β-Hippo signaling pathway. In addition, it is worth noting that compared with normal cells, the key biochemical feature of malignant tumor cells is the conversion of energy metabolism from oxidative phosphorylation to aerobic glycolysis. The excessive lactate production by cancer cells promotes tumor progression and is associated with tumor metastasis, angiogenesis, recurrence, treatment resistance and poor prognosis [40, 41]. Our study also demonstrated that genes related to lactate metabolism significantly affect the prognosis of OV.

However, this study has the following shortcomings to be further improved. First of all, the analysis of this study was carried out based on the information of limited clinical samples in the public database, and further expansion of the sample size would be an immediate concern for us. Secondly, the results of this study need to be further investigated by in vivo molecular experiments and the validity and clinical value of the prognostic model and nomogram need to be assessed in clinical practice. In addition, the prognostic value of metabolism-related genes in OV and their potential molecular mechanisms need to be further investigeted.

## Conclusion

In summary, this study divideded OV into two subtypes based on lactate metabolism-related genes and constructed a prognostic model of OV by identifying differentially expressed genes related to OV progression, providing potential for its clinical diagnosis and patient prognosis. Additionally, the functional enrichment analysis of the tumor microenvironment and differentially expressed genes in the high and low-risk groups further demonstrated the molecular mechanism of OV. In future, we can fully illustrate the molecular mechanisms of these biomarkers by further predicting their upstream miRNAs, downstream lncRNAs, potential TFs, and so on.

## Materials and methods

### Data source

The RNA-seq expression matrix data (updated to July 20, 2019), survival information, and clinical information of 354 OV samples were acquired from the TCGA database (https://xenabrowser.net, accessed 20 July 2019) which of 353 cancer samples contained survival and clinical information. The GSE66957, GSE119054 and GSE26712 datasets were acquired from the GEO database (https://www.ncbi.nlm.nih.gov/, assessed 1 March 2022). The GSE66957 (57 cancer and 12 normal samples) dataset and GSE119054 (6 cancer and 3 normal samples) dataset were used to perform differential expression analysis between cancer and normal samples. The GSE26712 dataset containing 153 cancer samples with survival information was used as an external validation set for the survival risk-scoring model. The “lactate metabolism-related genes” was used as the keyword to search for LMRGs in the MSigDB database (http://www.gsea-msigdb.org, 3 March 2022). A total of 15 LMRGs were obtained from the lactate metabolism-related pathway (LACTATE_METABOLIC_PROCESS) in the c5.go.bp.v7.4.symbols.gmt background gene set, including PARK7, LDHD, PNKD, HAGH, HIF1A, LDHA, LDHC, MIR210, PFKFB2, C12orf5 (TIGAR), MRS2, TP53, SLC25A12, PER2 and ACTN3.

### Consistent cluster analysis

Based on the expression matrix of LMRGs, the ConsensusClusterPlus package [42] was utilized to perform consistent clustering analysis on OV patients, and the best cluster method was selected by combining the front points with the largest changes in the CDF value and the downward trend of CDF. K-M curves of patients with different clusters were plotted to compare the survival probability using the survival [43]. The expression levels of LMRGs among different clusters were compared and visualized by Wilcoxon. Test and ggplot2 package [44].

In order to understand the correlation between different clusters and clinical characteristics, chi-square test was utilized to compared the differences in the percentage of sub-types with different clinical characteristics (age, OS, clinical stage, neoplasm cancer status, neoplasm histologic grade, and tumor residual disease) among clusters.

### Analysis of immune infiltration among different clusters

The microenvironment cell populations counter (MCPcounter) algorithm and the Single-sample gene set enrichment analysis (ssGSEA) were utilized to analyze the immune infiltration of patients with different clusters. The R package ggplot2 was used to draw a boxplot and the Wilcox.test method was used to screen out the immune cells with differences between different clusters.

### Differential gene analysis

The limma package [45] was applied to analyze the DEGs between clusters (cluster2 VS cluster1). Moreover, the DEGs between the cancer samples and normal samples in the GSE66957 and GSE119054 datasets (Tumor VS Normal) were acquired by limma package with P.Value < 0.05 & |log2fold change (FC)| > 0.5 as the threshold. The online tool Venn was applied to intersect the DEGs of the cancer and control samples in the GSE66957 and GSE119054 datasets. Furthermore, the intersection of the differentially intersecting genes of Tumor vs. Normal and the differential genes between the above clustering clusters were obtained.

### Construction and validation of prognostic model

Based on the intersection of DEGs obtained above, the step function in the survival package was applied to perform multivariate Cox analysis with the parameter direction = “both” to obtain the prognostic genes.Depending on the median value of risk score acquired by the following formula, the OV patients were separated into high- and low-risk groups.

$$risk \;score=\sum _{i=1}^{n}\left(\beta i*Xi\right)$$

In this formula, βi refered to the regression coefficient and X refered to the expression value of the gene. Subsequently, the K-M and ROC curves were painted by survminer package [46] and survivalROC package [47], respectively. Additionally, the prognostic model was validated in the GSE26712 dataset.

### Correlation analysis between risk models and clinical characteristics

The ggplot2 was applied to show the difference of risk scores in different clinical characteristics (age, OS, clinical stage, neoplasm cancer status, neoplasm histologic grade, tumor residual disease and cluster subtype) accessed by Wilcoxon.test method.

### Independent prognostic analysis of risk models and construction of nomogram

The clinicopathological factors of 353 cancer samples in the TCGA dataset were included in univariate Cox analysis to explore the independent prognosis of risk models and clinicopathological factors. The independent factors with *p* < 0.05 were included in the multivariate Cox analysis to obtain independent prognosis factors. In addition, the R package RMS [48] was applied to construct a nomogram to predict the 1/3/5-year survival probability of OV patients, and the calibration curve and ROC curve were drawn to verify the validity of the nomogram.

### Immune-related analysis

The ssGSEA algorithm [49] was used to analyze the infiltertion of 28 types of immune cells and 13 types for the patients in two risk groups, and the ggplot2 was employed to draw a boxplot. The Wilcoxon.test method was employed to screen the differences in immune cells and immune functions between the risk groups.

The expression of immune checkpoints (VTCN1, BTLA, CXCL9, CTLA4, IDO1, LAG3, CXCL10, IFNG, CD27, PDCD1, CD274 and HAVCR2) were extracted from the training set, and the immunotherapy response prediction analysis of patients in the risk groups were performed. Then, the R package ggplot2 was used to draw boxplots using the Wilcox.test method to show the differential immune checkpoints and TIDE scores between the risk groups.

#### Functional enrichment analysis

The limma was applied to analyze the DEGs of high- and low-risk samples in the gene expression matrix, and the corresponding P.Value and logFC values were obtained. The DEGs screening condition was P.Value < 0.05 & |log2 FC| > 0.5. The R package clusterProfiler [50] was employed to analyze the BP in GO and KEGG enrichment analysis of the above DEGs involved. Moreover, the GSEA software (V4.0.3) was used to obtain the pathways or functions involved in genes that differ between the two phenotypes.

### qRT-PCR

Based on 8 pairs of cryopreserved tissue samples, qRT-PCR was performed to validate the expression of 6 prognostic genes (CCL18, CCND1, MXRA5, NRBP2, OLFML2B and THY1). These samples were committed by the patients and the Ethics Committee of Shanxi Provincial People’s Hospital. Total RNA was extracted using TRIZol (Thermo Fisher, Shanghai, CN), and mRNA was reverse transcribed into cDNA sing SureScript-First-strand-cDNA-synthesis-kit (Servicebio, WuHan, CN). The qRT-PCR reaction system was made up of 3ul of cDNA, 5ul of 2xUniversal Blue SYBR Green qPCR Master Mix and 1ul of each upstream and downstream primers. Finally, the reactions were performed on a CFX96 real-time quantitative fluorescence PCR instrument. The amplification reactions were programmed with pre-denaturation at 95 °C for 1 min, followed by 40 cycles, each cycle consisting of 95 °C for 20 s, 55 °C for 20 s, and 72 °C for 30 s. The relative expression of genes was calculated by the 2^−ΔΔCt^ method using GAPDH as the internal reference gene. Primers for prognostic genes were shown in Table 1.

**Table 1:** The sequence of primers

Primer	Sequence
CCL18 forward	CCTGGCAGATTCCACAAAAGTT
CCL18 reverse	TAGGAGGATGACACCTGGCTTG
CCND1 forward	CAATGACCCCGCACGATTTC
CCND1 reverse	CATGGAGGGCGGATTGGAA
MXRA5 forward	TATCAACACCCTCTTCCGACC
MXRA5 reverse	CATGAACTCTTCCATCCTGGC
NRBP2 forward	GTGGACCACCCGAACATCG
NRBP2 reverse	CCTGATGACACGTACTCTGTGAT
OLFML2B forward	GACAAGGTCAAGGCTATGTCTG
OLFML2B reverse	TGGTTTCCACGGTATAGAAGTCT
THY1 forward	CTAACGGCCTGCCTAGTGGA
THY1 reverse	GGTTCGGGAGCGGTATGTGT
internal reference GAPDH forward	ACAACTTTGGTATCGTGGAAGG
internal reference GAPDH reverse	GCCATCACGCCACAGTTTC

## Data Availability

The datasets generated during the current study are available in TCGA (https://xenabrowser.net), GEO (https://www.ncbi.nlm.nih.gov/, accession number: GSE66957, GSE119054, and GSE26712), and MSigDB (http://www.gsea-msigdb.org) repositories.

## Abbreviations

LMRGs: lactate metabolism-related genes
OV: ovarian cancer
TCGA: The Cancer Genome Atlas
GEO: Gene Expression Omnibus
MSigDB: Molecular Signatures Database
DEGs: differentially expressed genes
K-M: Kaplan-Meier
ROC: receiver-perating characteristic
qRT-PCR: quantitative Real-Time Polymerase Chain Reaction
HGSC: high-grade serous carcinoma
CDF: cumulative distribution function
OS: overall survival
ssGSEA: Single-sample gene set enrichment analysis
MCPcounter: microenvironment cell populations counter
FC: fold change
KEGG: Kyoto Encyclopedia of Genes and Genome
BP: biological process
GO: Gene Ontology
